# Potential Applications of Artificial Intelligence in Clinical Trials for Alzheimer’s Disease

**DOI:** 10.3390/life12020275

**Published:** 2022-02-12

**Authors:** Younghoon Seo, Hyemin Jang, Hyejoo Lee

**Affiliations:** Samsung Alzheimer Research Center, Samsung Medical Center, 81 Irwon-ro, Gangnam-gu, Seoul 06351, Korea; young20021118@gmail.com (Y.S.); hmjang57@gmail.com (H.J.)

**Keywords:** Alzheimer’s disease, artificial intelligence, clinical trials, eligibility assessment, randomization

## Abstract

Clinical trials for Alzheimer’s disease (AD) face multiple challenges, such as the high screen failure rate and the even allocation of heterogeneous participants. Artificial intelligence (AI), which has become a potent tool of modern science with the expansion in the volume, variety, and velocity of biological data, offers promising potential to address these issues in AD clinical trials. In this review, we introduce the current status of AD clinical trials and the topic of machine learning. Then, a comprehensive review is focused on the potential applications of AI in the steps of AD clinical trials, including the prediction of protein and MRI AD biomarkers in the prescreening process during eligibility assessment and the likelihood stratification of AD subjects into rapid and slow progressors in randomization. Finally, this review provides challenges, developments, and the future outlook on the integration of AI into AD clinical trials.

## 1. Introduction

Recent advances in understanding the neurobiology of Alzheimer’s Disease (AD) revealed that the initiation of disease processes leading to symptomatic and functional neurodegeneration precedes the onset of dementia by 15–20 years [1,2]. AD is pathologically characterized by the aggregation of beta-amyloid (Aβ) plaques and hyperphosphorylated tau proteins in the form of neurofibrillary tangles (NFTs). The amyloid cascade hypothesis explains that Aβ triggers the following procession, such as the development of NFTs, cortical atrophy, cognitive impairments, and loss of activities of daily living [3,4,5]. These AD biomarkers appear in the predementia stage, including normal cognition (NC) and mild cognitive impairment (MCI). Thus, previous clinical trials have focused on the development of Aβ targeting diagnostic and therapeutic methods. There are also growing clinical trials targeting tau and NFTs, as tau pathology is more closely correlated with cognitive decline than Aβ [6].

Despite the stagnancy in AD clinical trials for the past 18 years ever since memantine was launched in 2003, a recent clinical trial of Biogen’s aducanumab has demonstrated a statistically significant reduction in Aβ plaques [7,8]. The US Food and Drug Administration has approved aducanumab for AD treatment using the Accelerated Approval pathway, which is expected to serve as an impetus for global AD clinical trial efforts. AD clinical trials involve two notable steps: eligibility assessment and randomization. During eligibility assessment, recruited participants are screened for either enrollment or exclusion, and, during randomization, selected participants are allocated into intervention and control groups. However, there are several challenges present in these steps. Principally, AD clinical trials have a high screen failure rate, which could be attributed to the stringent screening criteria of AD trials, such as Aβ PET positivity. Secondary and tertiary prevention trials for AD have average screen failure rates of 88% and 44%, respectively, which would suggest the need for considerable work to recruit even one eligible subject due to the expensive and time-consuming nature of screening procedures [9]. Furthermore, given the heterogeneous rates of AD progression, it is important to allocate subjects evenly into intervention and control groups based on their AD trajectories for a reliable observation of the treatment [10,11].

Artificial Intelligence (AI) refers to “the ability of a digital machine or computer to accomplish tasks that traditionally required human intelligence.” [12] A convergence of advanced AI algorithms, data proliferation, tremendous increases in computing power, and memory storage has propelled AI from hype to reality. ML algorithms could enhance the ability to detect hidden structures or underlying patterns of the data to improve the performance over time and learn how to make a prediction rather than explicit instruction. In this review, we aim to explore the applications of AI for a specific domain in clinical trials for AD in the steps of eligibility assessment and randomization. Finally, the development of explainable AI techniques and rigorous external validations cohorts with greater diversity would substantially benefit AD clinical trials.

## 2. Databases and Performance Measurements

Before discussing the applications of AI in AD clinical trials, we provide general information about the databases and performance measurements used by studies in this review.

### 2.1. Databases

Most studies tended to develop models based on established AD databases. The three most widely used databases are covered in Table 1.

Alzheimer’s Disease Neuroimaging Initiative (ADNI) is a multisite, longitudinal study that aims to develop and validate clinical, cognitive, imaging, genetic, and protein AD biomarkers (http://adni.loni.usc.edu, accessed on 4 February 2022). Open Access Series of Imaging Studies (OASIS) is another longitudinal neuroimaging, clinical, and cognitive biomarker dataset for normal aging and AD (https://www.oasis-brains.org, accessed on 4 February 2022). Lastly, AddNeuroMed (ANM) is a longitudinal European dementia cohort for AD biomarkers [13].

### 2.2. Performance Measurements

Many performance metrics were used to evaluate and compare their classification performance. Some commonly used measurements included Accuracy (ACC), Specificity (Spec), Sensitivity (Sens), classification error rate, Root mean squared error (RMSE), and area under the receiver operating characteristic curve (AUROC or AUC).

To evaluate AI-based synthetic PET images, several measurements were suggested. Maximum mean discrepancy (MMD) measures the distance between real and synthetic PET data distribution [14]. Structural similarity metric (SSIM) assesses the diversity of generated results by finding similarities within pixels of real and synthetic images [15]. Peak signal to noise ratio (PSNR) compares real and synthetic images using the ratio between the maximum possible intensity value and the mean squared error [16].

## 3. Eligibility Assessment

**Figure 1:**
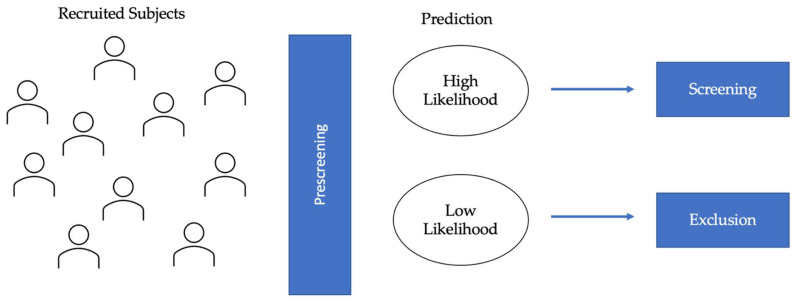
Diagram of eligibility assessment in AD clinical trials. AI-applications in eligibility assessment would prescreen the recruited subjects to identify the high likelihood and low likelihood groups. AI algorithms would be used to classify individuals based on predicted protein (Aβ and tau) biomarkers and/or MRI biomarkers. The high likelihood group would be selected for further screening, and the low likelihood group would be excluded, thereby leading to lower screen failure.

Screening is an important process in AD clinical trials to ascertain that selected participants are only those with AD pathology. Clinical diagnosis of AD follows the 1984 NINCDS-ADRDA Work Group criteria [17] or the 2011 NIA-AA guidelines [18]. Recent studies have shown that 15–25% of clinically diagnosed AD patients showed incompatible amyloid positron emission tomography (PET) or cerebrospinal fluid (CSF) findings [19,20]. Additionally, the increasing tendency of AD clinical trials to target preclinical stages, where cognition and functionality are normal, has underscored the importance of biomarker-guided screening. However, AD clinical trials have a high screen failure rate and corresponding low recruitment rate, as only one-third of asymptomatic older adults are Aβ+ [21]. Therefore, prescreening algorithms using AI could help reduce screen failure rate by classifying the recruited population into high and low likelihood groups, with the former undergoing screening procedures for validation and the latter being excluded from the clinical trials (Figure 1). This consideration applies to clinical trials for both disease-modifying therapies (DMTs) and symptomatic treatments.

### 3.1. Protein Biomarkers for AD

Although causal mechanisms remain unclear, Aβ and tau proteinopathies are defining features of AD as a unique disease [22]. AI prescreening algorithms can reduce challenges of PET and CSF, such as high costs and participants’ fear of radiation exposure, by selecting a subset of individuals who are likely to be Aβ or tau positive. Therefore, in AI research for AD clinical trials, many aimed to predict amyloidosis in subjects with MCI [23,24,25], while others focused on preclinical stages before neurodegeneration is too substantial [26,27] (Table 2).

Studies have suggested neuroimaging modalities as good predictors for Aβ+ status. One group proposed the least absolute shrinkage selection (LASSO) regression method to predict Aβ+ in 440 aMCI subjects [28]. Radiomics features were extracted from MRI images with hippocampus and precuneus as regions of interest (ROIs) and were used alone or in combination with baseline non-imaging predictors. Combining the T1 and T2 features (AUC: 0.75) improved the prediction from models only using either T1 (AUC: 0.71) or T2 (AUC: 0.74). A recent study classified a heterogeneous cohort of 337 NC, 375 MCI, and 98 AD using a support vector machine (SVM) [29]. The model was trained jointly using demographic information, neuropsychological (NP) test scores, *APOE* ε4 genotype, and MRI measures. For NC, an accuracy of 0.68 was achieved (Sens: 0.61; Spec: 0.71), MCI 0.75 (Sens: 0.71; Spec: 0.77), and whole sample 0.77 (Sens: 0.75; Spec: 0.79). Another study applied an ML classifier called longitudinal voxel-based classifier on the Jacobian determinant maps to detect abnormal Aβ levels in NC subjects [30]. In tensor-based morphometry, a Jacobian determinant map encodes the local volume difference between a reference and a target image [31]. Thus, the classifier (AUC: 0.87) relied on the local tissue change between Aβ+ and Aβ− NC subjects for classification. While MRI is the most popular modality, some studies have used other modalities, such as diffusion tensor imaging (DTI), which assesses the structural integrity of white matter tracts, could complement MRI to measure brain atrophy [32]. A multiple kernels SVM (MK-SVM) provided an accuracy of 66–68% for discrimination in identifying MCI Aβ+ vs. MCI Aβ− and 67–74% for MCI Aβ+ vs. NC.

Another research focus is on assessing Aβ status with non-imaging variables, which could reduce cost and save time. One group developed step-wise hierarchical regression models to investigate the relationship between the individual cognitive measure and Aβ in 41 aMCI subjects [33]. Story recall was highly accurate in predicting the Aβ burden (AUC: 0.86) and accounted for variance effects of age, education, hippocampal volume, and global cognition. A recent study evaluated the positive predictive value (PPV) of demographic, *APOE*, and cognitive information in the prediction of amyloid pathology in older NC subjects [34]. The random forest (RF) model estimated a PPV of 0.65, which would reduce the number of subjects undergoing biomarker screening from 2451 to 1539 in a clinical trial aimed at recruiting 1000 Aβ+ subjects. Another study developed the Preclinical Amyloid Sensitive Composite (PASC) to detect the cognitive differences between Aβ+ and Aβ− NC subjects [35]. The Multiple Indicator Multiple Cause (MIMIC) model was used to compare the latent means in the cognitive domains of two groups, and the multivariate analysis of covariance (MANCOVA) was performed to see the score differences of NP tests pertaining to episodic memory and executive functions. The PASC scores were calculated using principal component analysis (PCA) to obtain the weight for each test score and achieved an AUC of 0.764 when applied with demographic measures.

A global effort is underway to establish various trial-ready registries like the Brain Health Registry (www.brainhealthregistry.org, accessed on 4 February 2022) to facilitate AD trial recruitment. Therefore, there are many algorithms that aim to evaluate online information for prescreening [36,37]. For instance, Extreme Gradient Boosting (XGBoost) [38] is a tree-based ML technique that gives larger weights to misclassified data points at each iteration. An XGBoost model achieved an AUC of 0.60 to 0.74, depending on the various combinations of feature vectors, such as demographics, APOE genotype, cognitive and functional measures from the Trial-Ready Cohort in Preclinical/Prodromal Alzheimer’s Disease (TRC-PAD) [39].

Many AI-guided diagnostic efforts focused on Aβ, given the evidence in support of the amyloid cascade hypothesis [3] and countless clinical trials for anti-amyloid agents [40,41,42]. However, recent findings suggested that tau pathology has more intimate links with AD-related cognitive impairment than Aβ pathology, suggesting the tantalizing potential for clinical trials targeting tau [43,44]. A study classified 64 prodromal AD patients through GBM and RF algorithms [45]. A combination of demographic variables, MCI diagnosis information, NP test scores, *APOE* genotype, and cortical thickness resulted in the highest performance for GBM (AUC: 0.86) and RF (AUC: 0.82). The relative feature importance, calculated through MDA or Gini index, showed that the most important features to classify tau positivity were the cortical thickness of parietal and occipital lobes and delayed word recall test score. Another study used a multi-class convolutional neural network (MC-CNN) to predict A/T/N staging of 2000+ ADNI cases with known A/T/N status based on structural MRI alone [46]. It predicted “A” at an overall accuracy of 88%, “T” at 89%, and “N” at 95%. The performance of the MC-CNN model could be potentially improved by including demographics and clinical measures.

AI algorithms help clinical trials address the challenge of missing PET imaging data by predicting the abnormal protein aggregation in the scan. However, one group designed a 3-dimensional CNN architecture to complete missing PET patterns with MRI data from ADNI [47]. Deep CNNs are a type of multi-layer model capable of capturing nonlinear mapping between inputs and outputs [48]. The proposed architecture achieved AUC of 0.69 for MCI vs. NC, 0.68 progressive MCI (pMCI, MCI subjects who progress to AD) vs. stable MCI (sMCI, MCI subjects who remains stable), and 0.89 AD vs. NC, outperforming other missing estimation methods, such as KNN and Zero methods. Interestingly, the predictive performance improved when PET and MRI data were used together (MCI vs. NC: 0.76; pMCI vs. SMCI: 0.68; AD vs. NC: 0.93).

Efforts to reconstruct AI-based synthetic images especially focused on the generative adversarial network (GAN). In the adversarial training process of the GAN, the Generator generates fake images, and the Discriminator distinguishes real images from fake images until the Generator produces images that the Discriminator can no longer distinguish [49]. One study reconstructed plausible PET images from a gaussian noise distribution (2048-dimensional noise), reporting MMD of 1.78 and SSIM of 0.53 [50]. Another study created synthetic PET images of patients in NC, MCI, and AD stages using deep convolutional GAN (DCGAN) [16]. DCGAN improves on the first GAN by ensuring a more stable training stage through measures, such as learnable upsampling and downsampling [51]. It achieved a PSNR of 32.83 and a mean SSIM of 77.48. Moreover, a 2D-CNN model using the axial, coronal, and sagittal slices of synthesized PET images classified NC and AD with an overall accuracy of 71.45%. Additionally, the cycle-consistent GAN (cGAN) to learn bi-directional mappings between PET and MRI scans [52]. Then a Landmark-based Multi-model Multi-Instance Learning (LM^3^IL) network was developed to learn and fuse discriminative features of MRI and PET for AD diagnosis. The mean PSNR value of synthetic PET images from the 3D-cGAN model was 24.49, and the LM^3^IL method achieved an accuracy of 92.50% (Sens: 89.94%; Spec: 94.53%) for AD vs. NC and 79.06% (Sens: 55.26%; Spec: 82.85%) for pMCI and sMCI, which had a superior performance than a single-model variant of the proposed LM^3^IL method that only used MRI.

Limitations of PET and CSF as general population-screening tools have prompted the search for alternative ways to predict disease progression from more accessible tissues, such as blood. From the Australian Imaging, Biomarkers and Lifestyle (AIBL) cohort, 176 blood analytes and two ratios (Innogenetics Aβ_1–40_/Aβ_1–42_ and Mehta Aβ_1–40_/Aβ_1–42_) were considered along with age, gender, APOE genotype, and years of education in variable selection and model generation to predict continuous standardized uptake value ratios (SUVR) values through RF analysis [53]. The model achieved an AUC of 0.88 (Sens: 0.80; Spec: 0.82). Meanwhile, blood metabolites have also garnered considerable interest as a potential molecular fingerprint of disease progression [54,55]. Deep Learning (DL) and XGBoost algorithms were trained with metabolite data derived from 242 NC and 115 AD subjects and produced AUC of 0.85 and 0.88, respectively. By comparison, CSF measures of amyloid, p-tau, and t-tau using XGBoost achieved AUC of 0.78, 0.83, and 0.87, respectively, which highlights the potential of blood-based biomarkers.

### 3.2. MRI Biomarkers

Within the A/T/N framework, neurodegeneration reflects downstream effects of molecular AD pathology, closely correlating with cognitive and functional decline [56,57]. Among many neuroimaging modalities, structural MRI (sMRI) is widely used as a surrogate marker for neurodegeneration due to its relative availability, low costs, and good diagnostic accuracy [58,59,60,61]. Therefore, many have used AI to capture the spatial patterns of atrophy in MRI data to enhance the linkage between neurodegeneration and AD-related changes, which are shown in Table 3. MRI can reveal the anatomical differences between AD and NC to classify subjects into different stages of AD [62,63,64]. It can also detect MCI subjects who will convert to AD based on the temporal link between MRI abnormalities and the onset of cognitive impairment [65,66,67]. MCI subjects who convert to AD during the duration of the study are often categorized as pMCI, while those who remain in MCI or revert to NC are categorized as sMCI.

The hippocampus, one of the earliest areas to degenerate structurally, is a good indicator for detecting AD progression in stages before initial clinical expression [68,69]. One study modeled the shape of the hippocampus using spherical harmonics (SPHARM) coefficients, which were later used as features in a Radial Basis Functions kernels SVM (RBF-SVM) classifier [70]. It discriminated 25 elderly NC controls from 23 AD subjects with 94% accuracy (Sens: 96%; Spec 92%) and from 23 aMCI subjects with 83% accuracy (Sens: 83%; Spec: 84%). Another study proposed a fully automatic segmentation method of the hippocampus using anatomical and probabilistic information [71]. KNN algorithm was used to assign 605 ADNI subjects to the group whose mean was closest to the hippocampal volume of the participant; 76% of AD subjects and 71% of MCI subjects were correctly classified with respect to NC controls. One group compared the classification performance of SVM, artificial neural network (ANN), and Naïve Bayes (NB) classifiers [72]. Naïve Bayes classifiers are Bayes’ theorem-based classifiers robust to irrelevant attributes and with strong independence assumptions [73]. The hippocampus was segmented into seven subfields using an atlas-based automatic algorithm based on Markov random fields in FreeSurfer. They showed that the classification performances of hippocampal subfields volumes across three classifiers (SVM: 0.71; ANN: 0.73; NB: 0.69) were more accurate than the classification performances of whole hippocampal volume (SVM: 0.66; ANN: 0.67; NB: 0.65) in the individual classification of pMCI and sMCI.

Considerable research has also focused on the relationship between in vivo cortical thickness measurements and AD neuropathology in asymptomatic subjects [74]. A study identified specific patterns of cortical atrophy at four time periods to subdivide the pMCI subjects based on “time to conversion” [75]. It built four stratified linear discriminant analysis (LDA) classifiers and increased classification performance by avoiding double dipping. When compared to sMCI, 80.9% accuracy was achieved with the pMCI6 classifier (converted to AD in 6 months), 74.5% with pMCI12, 73.0% with pMCI24, and 77.3% with pMCI36. Another study developed a spatial frequency representation of cortical thickness data for classification based on incremental learning [76]. Cortical thickness data were mapped onto a spatial frequency domain with the manifold harmonic transform from the surface of the cortex. The PCA-LDA classifier discriminated NC from AD (Sens: 82%; Spec: 93%) and pMCI to sMCI (Sens: 63%; Spec: 76%). A group improved their classification based on cortical thickness features by combining SVM and AdaBoost [77]. AdaBoost iteratively increases the weights of misclassified samples and decreases the weights of correctly classified ones to combine multiple “weak classifiers” into a single “strong classifier.” The proposed method discriminated AD and NC with 84.38% accuracy, 4–10% higher than classical methods, such as SVM, LDA, and Gaussian mixture model (GMM). Beyond regional analysis, inter-regional covariation of cortical thickness has also been suggested for prognostic applications [78].

Other research focused on merging different biomarkers. Manual hippocampal volume measurement and automated global and regional volume measures were combined for the orthogonal partial least squares to latent structures (OPLS) analysis [79]. The combination of volume measures for AD vs. NC (Sens: 90%, Spec: 94%) resulted in higher sensitivity and specificity than hippocampal volume alone (Sens: 87%, Spec: 90%). NC vs. AD classification could also be performed through simultaneous patched-based segmentation [80]. The study segmented and graded the anatomical structures of the hippocampus and entorhinal cortex and used hippocampal and entorhinal volumes and grades (similarity of the patch surrounding the vortex), as well as their combinations, to find atrophic patterns. With LDA and quadratic discriminant analysis (QDA) as classifiers, hippocampal measures had more discriminating power than entorhinal measures, and 90% accuracy was achieved (which increased to 93% after adding the ages of subjects). Another study employed a greedy score-based feature selection technique to select important feature vectors (cortical thickness, surface area, folding indices, curvature indices, and volume) [81]. A regularized extreme learning machine (RELM) classifier, a type of learning algorithm implemented without iteratively tuning the artificial hidden nodes, achieved an accuracy of 57.56–61.20% for multi-class (AD, MCI, and NC) differentiation, higher than SVM (52.63–57.40%) and import vector machine (IVM) (54.90–55.50%).

Conventional ML, such as SVM, relies on laborious brain segmentation that requires complex image preprocessing techniques [82]. This challenge is addressed by DL approaches [83], which discover intricate structures in data without requiring prior feature selection or data preprocessing. A study developed 3D-CNNs whose first layer used filters learned with autoencoders [84]. The 3D-CNNs outperformed their 2D counterparts in 3-way (AD, MCI, and NC) and binary classifications (4–10%). Another study employed GoogLeNet [85] and Residual Network (ResNet) [86], the winners of ILSVRC in 2014 and 2015, respectively, for 4-way classification of AD, MCI, late MCI (lMCI), and NC [87]. Both GoogLeNet (99%) and ResNet (98%) achieved high accuracy. A recent study also proposed a deeply supervised adapTable 3 D-CNN (DSA-3D-CNN), with transfer learning for AD diagnosis [88]. The proposed classifier was built by stacking pre-trained 3D convolutional autoencoding layers followed by fully connected layers, which are fine-tuned for task-specific classification. It achieved accuracies from 94.2% to 100% in target domains (AD vs. MCI vs. NC, AD + MCI vs. NC, AD vs. NC, AD vs. MCI, and MCI vs. NC).

Ensemble-based classifiers [89] integrate individual decisions of multiple models to classify a new sample based on voting. A framework that ensembles three deep CNNs, each with slightly different configurations, was provided using the OASIS database [90]. Contrary to many existing approaches focusing on binary classification, the ensemble system classifies individuals into NC, very mild, mild, and moderate AD with an average precision of 94%. An ensemble of 3D densely connected convolutional networks (3D-DenseNets) was also proposed for AD and MCI diagnosis [91]. Dense connections were introduced to maximize information flow and improve feature utilization; with a dense connection mechanism, fewer feature increments are added to each layer, which reduces the number of parameters. It reached an accuracy of 0.9477 for NC vs. MCI vs. AD.

Most DL research uses CNNs, which effectively find highly layered features and tune hyperparameters [92]. Nevertheless, an early work in this area used a deep belief network (DBN) [93] for AD vs. NC classification [94]. Manifold learning was performed to reduce the dimensionality of 3D MRI images from ADNI by discovering patterns of similarity and variability. Another work proposed THS-GAN, i.e., Tensor-train decomposition, Higher-order pooling, and Semi-supervised learning were employed in the GAN model to assess MCI and AD using the ADNI data [95]. The tensor train decomposition is applied to all layers in the Generator and the Discriminator, which reduces the number of parameters. The higher-order pooling, compared to the first-order pooling, leverages the second-order statistics of the holistic MRI images, which effectively captures long-range dependencies between slices of different directions. Moreover, the model is designed in a semi-supervised manner to take advantage of both labeled and unlabeled MR images. With optimal hypermeter settings, THS-GAN reached accuracies of 95.92% (AD vs. NC), 89.29% (MCI vs. NC), and 85.71% (AD vs. MCI). While MRI is widely used as a surrogate marker for neurodegeneration, multimodal approaches combining various modalities, such as computer tomography (CT) and single-photon emission computerized tomography (SPECT), if available, can improve predictive performance [96].

## 4. Randomization

**Figure 2:**
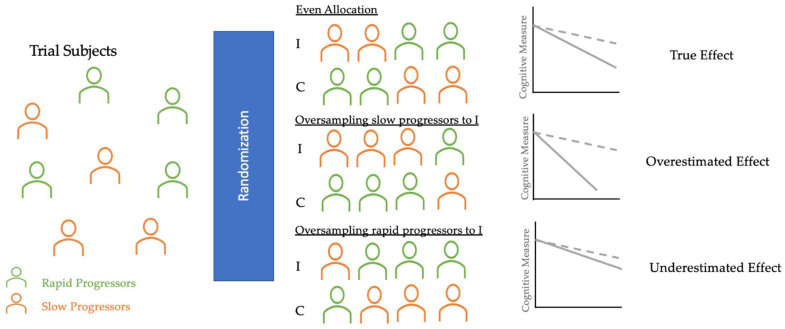
Diagram of randomization in AD clinical trials. These three potential scenarios of randomization illustrate how an uneven allocation of rapid or slow progressors (oversampling or undersampling) into I and C can obscure the true treatment effect. Thus, AI applications in randomization can classify trial subjects into these clusters and help trials achieve even allocation. *I*, Intervention group (dotted line); *C*, Control group (solid line).

Primary outcomes of AD clinical trials are often the absence of clinical progression (measured by scales, such as the Clinical Dementia Rating (CDR)) or cognitive deterioration (measured by NP test scores). However, many longitudinal studies have identified fast and slow AD progressors characterized by heterogeneous rates of cognitive and functional decline [97,98,99,100]. Randomization in clinical trials does not always allocate an equal proportion of rapid and slow progressors into control and intervention groups [101,102]. As Figure 2 shows, if rapid progressors are mainly selected for the intervention group and slow progressors for the control group, the reported treatment effect would seem as though the treatment had no significant impact, even if the treatment was, in fact, clinically efficacious. On the other hand, if slow progressors were mainly selected for the intervention and rapid progressors for the control group, the reported treatment would have overestimated the clinical efficacy. Therefore, an even allocation of rapid and slow decliners into intervention and control groups is desirable to reduce bias in treatment assignment and avoid the two aforementioned extreme scenarios that could explain the failures [103] and successes [104] of AD clinical trials in the last two decades.

For a reliable observation of the intervention impact, many have focused on predicting rapid progression using multimodal biomarkers (Table 4). A recent study performed a multivariable LR analysis to classify 124 Aβ+ MCI subjects, with rapid progressors defined as those who converted to AD status in 3 years of follow-up [105]. Univariate logistic analysis of rapid and slow progressors showed no significant differences in demographic measures, but the biomarker characteristics between the two differed significantly. Two separate analyses were conducted for CSF p-tau and CSF t-tau due to the multicollinearity between the variables, and the models achieved AUC of 0.901 and 0.907, respectively. LR models suggested that MCI status, *APOE4* status, corrected hippocampal volume (HV), [F18] fluorodeoxyglucose (FDG) PET SUVR, and CSF t-tau/p-tau were associated with fast AD progression, which was then used to construct nomograms where a specific point corresponds to each variable based on the beta coefficients of the regression analyses.

Another study used a DL model to differentiate rapid vs. slow progression in 321 ADNI subjects with baseline AT(N) biomarkers [106]. Rapid and slow progressors were first identified by applying an unsupervised time-series technique based on dynamic time-warping (DWT) [107] with Ward’s linkage-based agglomerative clustering [108], which allows for shape-based clustering of dynamic time-varying observations. These progression phenotypes were used to train Parameter-efficient Network Model (PENet) with baseline biomarkers for A (CSF Aβ 1–42), T (CSF p-tau 181), and N (MRI images and FDG-PET). PENet took a combination of AT(N) biomarkers to predict cognitive decline status, with a comprehensive AT(N) model (accuracy: 0.710) outperforming biomarker pairs (A(N): 0.683; T(N): 0.702) and individual biomarkers (A: 0.597; T: 0.567; N: 0.685), which suggested a synergistic relationship between these biomarkers.

Clustering algorithms are also a popular approach to identify homogeneous clusters of rapid and slow progressors. A multi-layer clustering (MLC) algorithm was proposed in a study to identify clusters of rapid and slow progressors among 562 ADNI MCI subjects [109]. The MLC model consisted of two steps: (1) example similarity tables were computed for each data layer, and (2) an agglomerative bottom-up procedure used these tables to find the optimal clustering solution. The subgroup discovery technique identified the best classifiers (clinical test cut-offs on Alzheimer’s Disease Assessment Scale (ADAS), MMSE, and Rey’s Auditory Verbal Learning Test (RAVLT)), and the classifiers achieved high sensitivity (75.0–98.4%) and specificity (70.0–90.0%). Findings showed that fast progressors had two-fold greater brain atrophy and converted to AD five times the rate of slow progressors. A hierarchical agglomerative clustering method was applied to MRI of a cohort of 751 MCI, 282 AD, and 428 NC [110]. The group preprocessed MRIs to gray matter density maps and regressed out age, gender, and years of education to render the maps comparable. The hierarchical clustering of MRIs discovered clusters of rapid and slow MCI progressors based on striking heterogeneities in brain atrophy patterns. Rapid progressors showed a higher degree of atrophy in the medial temporal lobe and cerebellum, while slow progressors manifested more atrophy in the frontal cortex.

Some groups have developed predictive algorithms without the use of PET and CSF. A ML classifier [76] trained with incremental learning was adopted to distinguish rapid and slow progressors in a longitudinal AD cohort [111]. The study used PCA for dimension reduction of the 27 high-resolution 3T brain MRIs and found coordinate axes that maximally separated the groups with LDA. It demonstrated that slow progressors showed discriminative patterns defined around the prefrontal and temporal cortices, while rapid progressors showed patterns in most of the prefrontal, inferior parietal, and temporal cortices. Another study used ADAS-Cog and MMSE, laboratory tests, and demographic information to train a Conditional Restricted Boltzmann Machine (CRBM) to forecast individual AD progression [112]. A CRBM [113] is a probabilistic neural network that learns from a joint probability distribution of features. Differences between rapid and slow progressors quantified using the absolute value of Cohen’s *d*-statistic showed that, while the majority of baseline features were not associated with rapid AD progression, strong associations were found with cognitive tests based on recall and word recognition. For instance, subjects with poor performance on the ADAS word recall tended to progress more rapidly. One group also reported using an unsupervised separation algorithm based on the genetic algorithm technique to uncover two distinct rates of AD progression characterized by the functional assessment staging (FAST) procedure [114].

Most research has investigated the heterogeneous progression based on clinical and functional measures, which are the primary outcomes in clinical trials. However, there are certain advantages of predicting rapid vs. slow biomarker progression. Firstly, AD clinical trials utilize biomarkers as secondary outcomes. Secondly, the knowledge of rapid and slow biomarker progression is important to correctly estimate the true effect size of anti-amyloid and other AD treatments. An uneven allocation of rapid and slow Aβ progressors could either dilute or exaggerate the clinical efficacy of the treatment. One group combined the baseline clinical, genetic, and imaging features from 610 unique ADNI subjects to identify those at the highest risk of rapid Aβ deposition [115]. They used a CNN based on the ResNet architecture [116] to identify important baseline amyloid PET image features, which were combined with eight clinical, demographic, and genetic markers to predict future SURVR values using a gradient-boosted decision tree (GBDT) algorithm. The combined model achieved a RMSE of 0.0339, which outperformed multivariate linear regression (0.0382) and GBDT without imaging features (0.0355). Moreover, the highest percentage of fastest Aβ progressors was predicted with the proposed method (37.7%), superior to other selection methods, such as Aβ+ cases with at least one *APOE* ε4 allele (15.7%).

## 5. Challenges and Future Directions

AI systems with large-scale data have facilitated the development of disease prediction that can potentially reduce the screen failure rate of clinical trials [39]. Furthermore, identifying suitable participants in trial recruitment contributes to reducing associated expenses and accelerates drug developments [117]. However, it is important to acknowledge several challenges for its applications in clinical trials.

Advanced AI models derived from high-quality databases often showed good predictive performance; additional information from explainable and transparent AI technology might further the understanding of biomedical data and improve their applications in clinical trials. A common form of a visible machine learning algorithm, such as a graphical neural network might provide structural connections between different medical entities (e.g., diseases, drugs, and proteins). For example, GNNexplainer identifies a small set of important variables and genetic pathways that contribute to human disease [118]. Identification of disease mechanisms through the multiscale interactome has facilitated efficacious and safe therapeutic development. In addition, earlier access to the drug candidates could help improve the time and expenditure of prescreening process in clinical trials. Thus, developing an explainable and transparent AI system would substantially benefit both the speed and efficiency of clinical trials and drug discovery.

Another challenge is the limited generalizability that arises from the lack of external validations, which reduces the confidence in the predictive power of AI algorithms. External and internal validations are crucial for the development of a reliable algorithm. Internal validation methods, such as bootstrap and cross-validation quantify the algorithm optimism and provide information about the degree of overfitting, whereas external validation uses independently derived data to ensure generalizability. Review studies, however, have demonstrated that a substantial number of studies did not perform any validation or performed either external or internal validation [119]. Internal validation methods could be limited by small sample size. A minimum of 300 subjects is generally recommended for internal validation, but categorical data in AD clinical trials, such as brain imaging measurements, could be limited due to the associated costs and time [120]. For external validation, over-reliance on one cohort population and unavailability of similar but the difference in cohort populations remains a challenge. These shortcomings limit the clinical relevance of AI despite its promising computational results.

Further studies need to focus on improving the efficiency and effectiveness of AI techniques for AD clinical trials. Firstly, AI technologies, such as visible neural networks, could incorporate the inner workings of AI models into complex and hierarchical biological systems [117,121]. AI models can be enriched with biological knowledge, which includes multilevel interactions composed of sequences, protein complexes, cells, tissues, organs, and organisms. Compared to the current deep learning schemes to model the entire system at once, this approach models how various AD-related entities interact with each other at different levels to develop the multiscale interactome for AD drug candidates. Moreover, these models could leverage genetic and genomic data to identify genetic determinants of AD to guide therapies with individuals’ genomic profiles, which allows for precision medicine and personalized treatment. Secondly, rigorous external validations in other populations with greater diversity are necessary to assess generalizability and reproducibility. Finally, given that the ML efficiency increases as the quantity and quality of data increases, the integration of genomics, proteomics, and other omics data in the AD clinical research could help investigate molecular pathways of AD, with potential implications for novel diagnostic biomarkers and precision medicine [122].

## 6. Conclusions

Clinical trials for AD face challenges of high screen failure and even allocation of the heterogeneous subject population. Many recent works have investigated the potential applications of AI to address these challenges in clinical trials, particularly in the steps of eligibility assessment and randomization. The prediction of protein and MRI AD biomarkers in the prescreening process could drastically reduce the high screen failure rate. Additionally, the AI-based stratification of the AD subject population into rapid and slow progressors can guide the even allocation of the heterogeneous AD population into intervention and control groups during randomization. AI algorithms have not been integrated into AD clinical trials due to the lack of explainability and poor external and internal validations. However, integrating biological knowledge to develop the multiscale interactome and rigorous external validations for generalizability and reproducibility could result in novel diagnostic biomarkers and precision medicine.

## Figures and Tables

**Table 1:** Summary of Widely Used AD Databases.

Name	Subjects	Modalities
ADNI	ADNI-1: 200 NC, 400 MCI, 200 mild AD ADNI-Go: 200 early MCI (eMCI) ADNI-2: 150 NC, 150 eMCI, 150 late MCI (lMCI), 200 mild AD ADNI-3: 135–500 NC, 150–515 MCI, 85–185 AD	MRI, PET, CSF, clinical/cognitive assessments, genetic data, blood biomarkers
OASIS	OASIS-1: 416 total, including 20 NC and 100 mild/moderate AD OASIS-2: 72 NC, 64 AD OASIS 3: 609 NC, 489 AD at various stages	MRI, PET, clinical and cognitive data
ANM	ANM: 266 NC, 247 MCI, 260 AD DCR: 423 NC, 89 MCI, 153 AD ART: 104 NC, 61 MCI, 99 AD	Clinical, proteomics, MRI, gene expression, genotype

DRC, Maudsley BRC Dementia Case Registry at King’s Health Partners cohort; ART, the Alzheimer’s Research Trust UK cohort.

**Table 2:** Summary of AI Algorithms for Protein Biomarkers.

Reference	Application	Method	Subjects	Performance
[26] 2012	Aβ+ vs. Aβ− NC	LR; demographic, family history, cognitive performance, *APOE*	483 NC	AUC: 0.62–0.70
[33] 2013	Aβ+ vs. Aβ− MCI	Step-wise hierarchical regression; cognitive measures, hippocampal atrophy, white matter hyperintensities (WMH)	41 aMCI	AUC: 0.86 (story recall)
[53] 2014	Aβ prediction	RF; blood and plasma analytes	169 NC, 55 MCI, 49 AD	AUC: 0.88; Sens: 80%; Spec: 82%
[47] 2014	PET image synthesis	3D-CNN; using MRI data	198 AD, 167 pMCI, 236 sMCI, 229 NC	AUC: 0.69 (MCI vs. NC), 0.68 (pMCI vs. sMCI), 0.89 (AD vs. NC)
[32] 2015	MCI Aβ+ vs. MCI Aβ−, MCI Aβ+ vs. NC	SVM; DTI and volumetric MRI data	25 NC, 35 Aβ− MCI, 35 Aβ+ MCI	Acc: 66–68% (MCI Aβ+ vs. MCI Aβ−), 67–74% (MCI Aβ+ vs. NC)
[23] 2015	Aβ+ vs. Aβ− MCI	Partial least squares (PLS); use anatomical shape variations from MRI	46 NC, 62 MCI	AUC: 0.70 (MRI), 0.81 (APOE), 0.88 (APOE + MRI)
[34] 2016	Aβ prediction	RF; demographics, *APOE*, cognitive rates	206 Aβ+, 125 Aβ−	PPV: 0.65
[52] 2018	PET image synthesis; AD vs. NC, pMCI vs. sMCI	3D-cGAN (using MRI); LM^3^IL for diagnosis	ADNI-1, ADNI-2	PSNR: 24.49; AD vs. NC: 92.50% (*Acc*), 89.94% (*Sens*), 94.53% (*Spec*); pMCI vs. sMCI: 79.06% (*Acc*), 55.26% (*Sens*), 82.85% (*Spec*)
[29] 2018	Aβ prediction	SVM; subcortical volumes, cortical thickness, and surface area	337 NC, 375 MCI, 98 AD	NC: 0.68 (*Acc*), 0.61 (*Sens*), 0.7 (*Spec*); MCI: 0.75 (*Acc*), 0.71 (*Sens*), 0.77 (*Spec*); whole: 0.77 (*Acc*), 0.75 (*Sens*), 0.79 (*Spec*)
[24] 2018	Aβ prediction in MCIAD	Multivariate stepwise LR; information commonly obtained in memory clinics	107 MCI, 69 AD	AUC: 0.873
[27] 2019	Aβ prediction in NC/MCI	RF; cognitive, genetic, and socio-demographic features	ADNI-MCI (596), ADNI-NC (318); INSIGHT (318)	AUC: 82.4% (ADNI-MCI), 69.1% (ADNI-NC), 67.5% (INSIGHT)
[30] 2019	Aβ prediction in NC	Longitudinal voxel-based classifier; Jacobian determinant maps	79 NC, 50 preclinical AD (PreAD), 274 MC/AD	AUC: 0.87
[46] 2019	A/T/N staging prediction	MC-CNN; sMRI	5000+ ADNI cases with known A/T/N staging	Acc: 88% (“A”), 89% (“T”), 95% (N)
[16] 2020	PET image synthesis; NC vs. AD classification	DCGAN; 2D-CNN using MRI and synthetic PET	98 AD, 105 NC, 208 MCI	PSNR: 32.83; SSIM: 77.48; Acc: 71.45% (NC vs. AD)
[50] 2020	PET image synthesis	GAN; gaussian noise distribution (2048-dimensional noise)	A subset of ADNI-1 with labled PET images	MMD: 1.78; SSIM: 0.53
[35] 2020	Aβ+ vs. Aβ− NC	PASC score using MIMIC and MANCOVA; NP scores	348 Aβ− NC, 75 Aβ+ NC	AUC: 0.764 (with demographic measures)
[36] 2020	Aβ prediction	LR; self-report information from BHR	70,992 subjects from BHR	Cross-validated AUC (cAUC): 0.62–0.66
[45] 2021	Tau prediction in prodromal AD	GBM and RF; combinations of clinical and NP data, cortical thickness	64 Aβ+ prodromal AD	AUC: 0.86 (GBM), 0.82 (RF)
[28] 2021	Aβ+ vs. Aβ− MCI	LASSO regression; using radiomics features extracted from T1 and T2 MRI	182 Aβ− MCI, 166 Aβ+ MCI	AUC: 0.75 (T1+T2), 0.71 (T1), 0.74 (T2)
[37] 2021	Aβ prediction	RF, SVM; combination of objective and subjective data from BHR	664 subjects from BHR	AUC: 0.519–0.624 (RF), 0.486–0.603 (SVM)
[55] 2021	AD vs. NC	DL, XGBoost; blood metabolites	242 NC, 115 AD	AUC: 0.85 (DL), 0.88 (XGBoost)

**Table 3:** Summary of AI Algorithms for MRI Biomarkers.

Reference	Application	Method	Subjects	Performance
[71] 2009	NC vs. MCI vs. AD	KNN; segmented hippocampus using anatomical and probabilistic priors	166 NC, 294 MCI, 145 AD	Classification rate: 76% (AD), 71% (MCI) with respect to NC
[70] 2009	NC vs. MCI vs. AD	RBF-SVM; model the shape of the hippocampus using SPHARM	25 NC, 23 aMCI, 23 AD	AD vs. NC: 94% (*Acc*), 96% (*Sens*), 92% (*Spec*); MCI vs. NC: 83% (*Acc*), 83%(*Sens*), 84% (*Spec*)
[79] 2011	AD vs. NC, AD vs. MCI, MCI vs. NC	OPLS analysis; hippocampal volume, regional and global volume measures	112 NC, 122 MCI, 117 AD	AD vs. NC: 90% (*Sens*), 94% (*Spec*) AD vs. MCI: 75% (*Sens*), 73% (*Spec*) MCI vs. NC: 66% (*Sens*), 73% (*Spec*)
[65] 2011	NC vs. AD; prediction of MCI to AD	SVM; 3D hippocampal morphology	88 NC, 103 MCI, 71 AD	AD vs. NC: 85% (*Acc*) MCI to AD: 80% (*Acc*), 77% (*Sens*), 80% (*Spec*)
[76] 2012	NC vs. AD; pMCI vs. sMCI	PCA-LDA; used manifold harmonic transform to represent cortical thickness data	160 NC, 131 sMCI, 72 pMCI, 128 AD	AD vs. NC: 82 (*Sens*), 93% (*Spec*) sMCI vs. pMCI: 63% (*Sens*), 76% (*Spec*)
[80] 2012	NC vs. AD	QDA and LDA; atrophic patterns of hippocampus and entorhinal cortex	60 NC, 60 AD	90% (*Acc*), 88% (*Sens*), 94% (*Spec*)
[75] 2013	pMCI vs. sMCI, NC vs. AD	LDA; ROI-wise patterns of cortical thinning	226 NC, 134 sMCI, 340 pMCI, 194 AD	Acc: 84.5% (AD vs. NC), 75.8% (sMCI vs. pMCI6), 72.9% (sMCI vs. pMCI12), 66.7% (sMCI vs. pMCI24), 69.9% (sMCI vs. pMCI36)
[77] 2013	NC vs. AD	SVM-based Adaboost; cortical thickness features	60 NC, 40 AD	Acc: 94.38%
[66] 2014	NC vs. AD; MCI to AD prediction	RF; combining cortical thickness and volumetric measures	225 NC, 165 MCI, 185 AD	AD vs. NC: 86.7% (*Acc*), 90.7% (*Sens*), 82.9% (*Spec*); MCI to AD: 78.0% (*Acc*)
[62] 2014	NC vs. AD	Ensemble of SVM, multi-layer perceptron (MLP), and decision tree (DT); volume of gray matter (GM), white matter (WM), CSF, and hippocampus area	NC: 48, AD: 37	93.75% (*Acc*), 100% (*Spec*), 87.5% (*Sens*)
[78] 2015	NC vs. AD, NC vs. pMCI, NC vs. MCI, pMCI vs. sMCI	Variational Bayes probabilistic multiple kernel learning (VBpMKL); inter-regional covariation of cortical thickness	159 NC, 56 pMCI, 130 sMCI, 136 AD	AUC: 0.92 (NC vs. AD), 0.83 (NC vs. pMCI), 0.75 (NC vs. MCI), 0.68 (pMCI vs. sMCI)
[84] 2015	NC vs. MCI vs. AD; NC vs. AD, MCI vs. AD, NC vs. MCI	3D-CNN combined with sparse autoencoders	755 subjects from ADNI; 2,265 scans	Acc: 89.47% (3-way), 95.39% (AD vs. NC), 86.84% (AD vs. MCI), 92.11% (NC vs. MCI)
[63] 2015	NC vs. AD; NC vs. MCI; AD vs. MCI	SVM; used circular harmonic functions on the hippocampus and posterior cingulate cortex	162 NC, 210 MCI, 137 AD	AD vs. NC: 83.77% (*Acc*), 88.2% (*Spec*), 79.09% (*Sens*); NC vs. MCI: 69.45% (*Acc*), 74.8% (*Spec*), 62.52% (*Sens*); AD vs. MCI: 62.07% (*Acc*), 75.15% (*Spec*), 49.02% (*Sens*)
[72] 2016	pMCI vs. sMCI	SVM, ANN, NB; hippocampal subfield atrophies	47 NC, 89 sMCI, 32 pMCI, 55 AD	SVM: 0.66 (*Acc*), 0.64 (*Sens*), 0.72 (*Spec*); ANN: 0.67 (*Acc*), 0.65 (*Sens*), 0.72 (*Spec*); NB: 0.65 (*Acc*), 0.63 (*Sens*), 0.72 (*Spec*)
[64] 2017	AD vs. NC, AD vs. eMCI, AD vs. lMCI, lMCI vs. NC, lMCI vs. eMCI, eMCI vs. NC	voxCNN, ResNet	61 NC, 77 eMCI, 43 lMCI, 50 AD	AD vs. NC AUC: 0.88 (VoxCNN), 0.87 (ResNet); AD vs. eMCI: 0.66 (VoxcNN), 0.67 (ResNet); AD vs. lMCI: 0.61 (VoxCNN), 0.62 (ResNet); lMCI vs. NC: 0.67 (Vox CNN), 0.65 (ResNet); lMCI vs. eMCI: 0.47 (VoxCNN), 0.52 (ResNet); eMCI vs. NC: 0.57 (VoxCNN), 0.58 (ResNet)
[87] 2017	NC vs. MCI vs. lMCI vs. AD	CNN-based architecture for GoogLeNet and ResNet	45 NC, 49 MCI, 22 lMCI, 33 AD (355 MRI volumes)	Overall Acc: 98.88% (GoogLeNet), 98.01% (ResNet-18), 98.14% (ResNet-152)
[81] 2017	NC vs. MCI vs. AD	SVM, IVM, RELM; using a greedy score-based feature selection	70 NC, 74 MCI, 70 AD	SVM Acc: 52.63–57.40%; IVM Acc: 54.90–55.50%; RELM Acc: 57.56–61.20%
[88] 2018	AD vs. MCI vs. NC, AD + MCI vs. NC, AD vs. NC, AD vs. MCI, MCI vs. NC	DSA-3D-CNN with transfer learning; modified for different domain	70 NC, 70 MCI, 70 AD (2265 scans total)	AD vs. NC Acc: 99.3; AD + MCI vs. NC Acc: 95.73; AD vs. MCI Acc: 100; and MCI vs. NC Acc: 94.22, AD vs. MCI vs. NC Acc: 94.8
[90] 2018	NC vs. very mild AD vs. mild AD vs. moderate AD	Ensemble of three 3D-CNNs	OASIS-1	Average precision: 0.94
[91] 2019	NC vs. MCI vs. AD	Ensemble of 3D-DenseNets	833 T1-weights MRIs from 624 ADNI subjects	Acc: 0.9477
[95] 2020	NC vs. AD, MCI vs. NC, AD vs. MCI	THS-GAN	221 AD, 297 MCI, 315 NC T1-MRI images	Acc: 95.92% (AD vs. NC), 89.29% (MCI vs. NC), 85.71% (AD vs. MCI)

**Table 4:** Summary of AI Algorithms for Rapid vs. Slow Progressor Prediction.

Reference	Application	Method	Subjects	Performance
[114] 2012	Rapid vs. slow AD disease courses	Separation algorithm based on genetic algorithm technique; FAST stage duration	Longitudinal course of 648 AD patients	FAST mean stage duration: 4: 1.60 (rapid), 3.17 (slow); 5: 0.71 (rapid), 2.26 (slow); 6: 1.69 (rapid), 3.30 (slow); 7: 5.24 (rapid), 6.73 (slow)
[109] 2017	Rapid vs. slow MCI	MLC; baseline and prognostic characteristics	562 MCI from ADNI-1 and ADNI-2	Sens: 75.0–98.4%, Spec: 70.0–90.0%
[111] 2018	Rapid vs. slow cognitive decline	PCA-LDA; cortical atrophy pattern	869 NC, 473 AD	*p* = 0.029 (significant different between rapid and slow progressors)
[105] 2019	Rapid vs. slow MCI	LR; CSF, FDG-PET, 18F-AV45 PET, hippocampal volume	186 MCI (74 rapid progressors, 112 slow progressors)	AUC: CSF p-tau model: 0.091; CSF t-tau model: 0.907
[112] 2019	Patient trajectory prediction	CRBM; using ADAS-Cog and MMSE, laboratory tests, and demographic information	1,909 subjects with MCI or AD	LR analysis: real and synthetic data were statistically indistinguishable
[110] 2020	Rapid vs. slow MCI	Hierarchical clustering; GM density maps	428 NC, 751 MCI, 282 AD	95% confidence intervals do not overlap
[115] 2021	Rapid vs. slow Aβ accumulation	GBDT; 18F-AV45 PET, clinical, demographic, and genetic markers	610 subjects with 1136 follow-up scans	RMSE: 0.0339; percentage of fastest Aβ progressors predicted (37.7%)
[106] 2022	Rapid vs. slow progression	PENet; using progression phenotype based on DWT	321 ADNI subjects	Acc: AT(N): 0.710; A(N): 0.683; T(N): 0.702; A: 0.597; T: 0.567; N: 0.675

## Data Availability

Not applicable.

## References

[1] Ritchie C.W., Molinuevo J.L., Truyen L., Satlin A., Van der Geyten S., Lovestone S., European Prevention of Alzheimer’s Dementia Consortium (2016). Development of interventions for the secondary prevention of Alzheimer’s dementia: The European Prevention of Alzheimer’s Dementia (EPAD) project. Lancet Psychiatry.

[2] Leal S.L., Lockhart S.N., Maass A., Bell R.K., Jagust W.J. (2018). Subthreshold Amyloid Predicts Tau Deposition in Aging. J. Neurosci..

[3] Karran E., Mercken M., De Strooper B. (2011). The amyloid cascade hypothesis for Alzheimer’s disease: An appraisal for the development of therapeutics. Nat. Rev. Drug Discov..

[4] Jack C.R., Knopman D.S., Jagust W.J., Petersen R.C., Weiner M.W., Aisen P.S., Shaw L.M., Vemuri P., Wiste H.J., Weigand S.D. (2013). Tracking pathophysiological processes in Alzheimer’s disease: An updated hypothetical model of dynamic biomarkers. Lancet Neurol..

[5] Hardy J.A., Higgins G.A. (1992). Alzheimer’s disease: The amyloid cascade hypothesis. Science.

[6] Giannakopoulos P., Herrmann F.R., Bussiere T., Bouras C., Kovari E., Perl D.P., Morrison J.H., Gold G., Hof P.R. (2003). Tangle and neuron numbers, but not amyloid load, predict cognitive status in Alzheimer’s disease. Neurology.

[7] Mangialasche F., Solomon A., Winblad B., Mecocci P., Kivipelto M. (2010). Alzheimer’s disease: Clinical trials and drug development. Lancet Neurol..

[8] Cummings J., Aisen P., Lemere C., Atri A., Sabbagh M., Salloway S. (2021). Aducanumab produced a clinically meaningful benefit in association with amyloid lowering. Alzheimer’s Res..

[9] Malzbender K., Lavin-Mena L., Hughes L., Bose N., Goldman D., Patel D., Leonard D. (2020). Key Barriers to Clinical Trials for Alzheimer’s Disease.

[10] DeCarli C. (2003). Mild cognitive impairment: Prevalence, prognosis, aetiology, and treatment. Lancet Neurol..

[11] Nettiksimmons J., Harvey D., Brewer J., Carmichael O., DeCarli C., Jack C.R., Petersen R., Shaw L.M., Trojanowski J.Q., Weiner M.W. (2010). Subtypes based on cerebrospinal fluid and magnetic resonance imaging markers in normal elderly predict cognitive decline. Neurobiol. Aging.

[12] Bali J., Bali O. (2021). Artificial intelligence in ophthalmology and healthcare: An updated review of the techniques in use. Indian J. Ophthalmol..

[13] Lovestone S., Francis P., Kloszewska I., Mecocci P., Simmons A., Soininen H., Spenger C., Tsolaki M., Vellas B., Wahlund L.O. (2009). AddNeuroMed—The European collaboration for the discovery of novel biomarkers for Alzheimer’s disease. Ann. N. Y. Acad. Sci..

[14] Gretton A., Borgwardt K.M., Rasch M.J., Scholkopf B., Smola A. (2012). A kernel two-sample test. J. Mach. Learn. Res..

[15] Odena A., Olah C., Shlens J. Conditional image synthesis with auxiliary classifier GANs. Proceedings of the 34th International Conference on Machine Learning.

[16] Islam J., Zhang Y. (2020). GAN-based synthetic brain PET image generation. Brain Inf..

[17] McKhann G., Drachman D., Folstein M., Katzman R., Price D., Stadlan E.M. (1984). Clinical diagnosis of Alzheimer’s disease: Report of the NINCDS-ADRDA Work Group under the auspices of Department of Health and Human Services Task Force on Alzheimer’s Disease. Neurology.

[18] McKhann G.M., Knopman D.S., Chertkow H., Hyman B.T., Jack C.R., Kawas C.H., Klunk W.E., Koroshetz W.J., Manly J.J., Mayeux R. (2011). The diagnosis of dementia due to Alzheimer’s disease: Recommendations from the National Institute on Aging-Alzheimer’s Association workgroups on diagnostic guidelines for Alzheimer’s disease. Alzheimer’s Dement..

[19] Landau S.M., Horng A., Fero A., Jagust W.J., Alzheimer’s Disease Neuroimaging Initiative (2016). Amyloid negativity in patients with clinically diagnosed Alzheimer disease and MCI. Neurology.

[20] Sevigny J., Suhy J., Chiao P., Chen T., Klein G., Purcell D., Oh J., Verma A., Sampat M., Barakos J. (2016). Amyloid PET Screening for Enrichment of Early-Stage Alzheimer Disease Clinical Trials: Experience in a Phase 1b Clinical Trial. Alzheimer Dis. Assoc. Disord..

[21] Chetelat G., La Joie R., Villain N., Perrotin A., de La Sayette V., Eustache F., Vandenberghe R. (2013). Amyloid imaging in cognitively normal individuals, at-risk populations and preclinical Alzheimer’s disease. Neuroimage Clin..

[22] Jack C.R., Bennett D.A., Blennow K., Carrillo M.C., Dunn B., Haeberlein S.B., Holtzman D.M., Jagust W., Jessen F., Karlawish J. (2018). NIA-AA Research Framework: Toward a biological definition of Alzheimer’s disease. Alzheimer’s Dement..

[23] Tosun D., Joshi S., Weiner M.W., Alzheimer’s Disease Neuroimaging Initiative (2013). Neuroimaging predictors of brain amyloidosis in mild cognitive impairment. Ann. Neurol..

[24] Lee J.H., Byun M.S., Yi D., Sohn B.K., Jeon S.Y., Lee Y., Lee J.Y., Kim Y.K., Lee Y.S., Lee D.Y. (2018). Prediction of Cerebral Amyloid With Common Information Obtained From Memory Clinic Practice. Front. Aging Neurosci..

[25] Shan G., Bernick C., Caldwell J.Z.K., Ritter A. (2021). Machine learning methods to predict amyloid positivity using domain scores from cognitive tests. Sci. Rep..

[26] Mielke M.M., Wiste H.J., Weigand S.D., Knopman D.S., Lowe V.J., Roberts R.O., Geda Y.E., Swenson-Dravis D.M., Boeve B.F., Senjem M.L. (2012). Indicators of amyloid burden in a population-based study of cognitively normal elderly. Neurology.

[27] Ansart M., Epelbaum S., Gagliardi G., Colliot O., Dormont D., Dubois B., Hampel H., Durrleman S., Alzheimer’s Disease Neuroimaging Initiative, INSIGHT-preAD Study (2020). Reduction of recruitment costs in preclinical AD trials: Validation of automatic pre-screening algorithm for brain amyloidosis. Stat. Methods Med. Res..

[28] Kim J.P., Kim J., Jang H., Kim J., Kang S.H., Kim J.S., Lee J., Na D.L., Kim H.J., Seo S.W. (2021). Predicting amyloid positivity in patients with mild cognitive impairment using a radiomics approach. Sci. Rep..

[29] Ten Kate M., Redolfi A., Peira E., Bos I., Vos S.J., Vandenberghe R., Gabel S., Schaeverbeke J., Scheltens P., Blin O. (2018). MRI predictors of amyloid pathology: Results from the EMIF-AD Multimodal Biomarker Discovery study. Alzheimer’s Res..

[30] Petrone P.M., Casamitjana A., Falcon C., Artigues M., Operto G., Cacciaglia R., Molinuevo J.L., Vilaplana V., Gispert J.D., Alzheimer’s Disease Neuroimaging Initiative (2019). Prediction of amyloid pathology in cognitively unimpaired individuals using voxel-wise analysis of longitudinal structural brain MRI. Alzheimer’s Res..

[31] Leow A.D., Yanovsky I., Chiang M.C., Lee A.D., Klunder A.D., Lu A., Becker J.T., Davis S.W., Toga A.W., Thompson P.M. (2007). Statistical properties of Jacobian maps and the realization of unbiased large-deformation nonlinear image registration. IEEE Trans. Med. Imaging.

[32] Dyrba M., Barkhof F., Fellgiebel A., Filippi M., Hausner L., Hauenstein K., Kirste T., Teipel S.J., EDSD Study Group (2015). Predicting Prodromal Alzheimer’s Disease in Subjects with Mild Cognitive Impairment Using Machine Learning Classification of Multimodal Multicenter Diffusion-Tensor and Magnetic Resonance Imaging Data. J. Neuroimaging.

[33] Bahar-Fuchs A., Villemagne V., Ong K., Chetelat G., Lamb F., Reininger C.B., Woodward M., Rowe C.C. (2013). Prediction of amyloid-beta pathology in amnestic mild cognitive impairment with neuropsychological tests. J. Alzheimer’s Dis..

[34] Insel P.S., Palmqvist S., Mackin R.S., Nosheny R.L., Hansson O., Weiner M.W., Mattsson N. (2016). Assessing risk for preclinical beta-amyloid pathology with APOE, cognitive, and demographic information. Alzheimer’s Dement..

[35] Hahn A., Kim Y.J., Kim H.J., Jang H., Cho H., Choi S.H., Kim B.C., Park K.W., Na D.L., Chin J. (2020). The preclinical amyloid sensitive composite to determine subtle cognitive differences in preclinical Alzheimer’s disease. Sci. Rep..

[36] Ashford M.T., Neuhaus J., Jin C., Camacho M.R., Fockler J., Truran D., Mackin R.S., Rabinovici G.D., Weiner M.W., Nosheny R.L. (2020). Predicting amyloid status using self-report information from an online research and recruitment registry: The Brain Health Registry. Alzheimer’s Dement..

[37] Albright J., Ashford M.T., Jin C., Neuhaus J., Rabinovici G.D., Truran D., Maruff P., Mackin R.S., Nosheny R.L., Weiner M.W. (2021). Machine learning approaches to predicting amyloid status using data from an online research and recruitment registry: The Brain Health Registry. Alzheimer’s Dement..

[38] Chen T., Guestrin C. XGBoost: A scalable tree boosting system. Proceedings of the 22nd ACM SIGKDD International Conference on Knowledge Discovery and Data Mining.

[39] Langford O., Raman R., Sperling R.A., Cummings J., Sun C.K., Jimenez-Maggiora G., Aisen P.S., Donohue M.C. (2020). Predicting Amyloid Burden to Accelerate Recruitment of Secondary Prevention Clinical Trials. J. Prev. Alzheimer’s Dis..

[40] Honig L.S., Vellas B., Woodward M., Boada M., Bullock R., Borrie M., Hager K., Andreasen N., Scarpini E., Liu-Seifert H. (2018). Trial of Solanezumab for Mild Dementia Due to Alzheimer’s Disease. N. Engl. J. Med..

[41] Salloway S., Farlow M., McDade E., Clifford D.B., Wang G., Llibre-Guerra J.J., Hitchcock J.M., Mills S.L., Santacruz A.M., Aschenbrenner A.J. (2021). A trial of gantenerumab or solanezumab in dominantly inherited Alzheimer’s disease. Nat. Med..

[42] Salloway S., Cummings J. (2021). Aducanumab, Amyloid Lowering, and Slowing of Alzheimer Disease. Neurology.

[43] Ossenkoppele R., Smith R., Ohlsson T., Strandberg O., Mattsson N., Insel P.S., Palmqvist S., Hansson O. (2019). Associations between tau, Abeta, and cortical thickness with cognition in Alzheimer disease. Neurology.

[44] Bejanin A., Schonhaut D.R., La Joie R., Kramer J.H., Baker S.L., Sosa N., Ayakta N., Cantwell A., Janabi M., Lauriola M. (2017). Tau pathology and neurodegeneration contribute to cognitive impairment in Alzheimer’s disease. Brain.

[45] Kim J., Park Y., Park S., Jang H., Kim H.J., Na D.L., Lee H., Seo S.W. (2021). Prediction of tau accumulation in prodromal Alzheimer’s disease using an ensemble machine learning approach. Sci. Rep..

[46] Lang A., Weiner M.W., Tosun D. (2019). What can structural MRI tell about A/T/N staging?. Alzheimer’s Dement..

[47] Li R., Zhang W., Suk H.I., Wang L., Li J., Shen D., Ji S. (2014). Deep learning based imaging data completion for improved brain disease diagnosis. Med. Image Comput. Comput. Assist. Interv..

[48] LeCun Y., Bottou L., Bengio Y., Haffner P. (1998). Gradient-based learning applied to document recognition. Proc. IEEE.

[49] Goodfellow I., Pouget-Abadie J., Mirza M., Xu B., Warde-Farley D., Ozair S., Courville A., Bengio Y. Generative adversarial nets. Proceedings of the Advances in Neural Information Processing Systems.

[50] Hu S., Yu W., Chen Z., Wang S. Medical image reconstruction using generative adversarial network for Alzheimer disease assessment with class-imbalance problem. Proceedings of the 2020 IEEE 6th International Conference on Computer and Communications (ICCC).

[51] Radford A., Metz L., Chintala S. (2015). Unsupervised representation learning with deep convolutional generative adversarial networks. arXiv.

[52] Pan Y., Liu M., Lian C., Zhou T., Xia Y., Shen D. (2018). Synthesizing Missing PET from MRI with Cycle-consistent Generative Adversarial Networks for Alzheimer’s Disease Diagnosis. Med. Image Comput. Comput. Assist. Interv..

[53] Burnham S.C., Faux N.G., Wilson W., Laws S.M., Ames D., Bedo J., Bush A.I., Doecke J.D., Ellis K.A., Head R. (2014). A blood-based predictor for neocortical Abeta burden in Alzheimer’s disease: Results from the AIBL study. Mol. Psychiatry.

[54] Whiley L., Legido-Quigley C. (2011). Current strategies in the discovery of small-molecule biomarkers for Alzheimer’s disease. Bioanalysis.

[55] Stamate D., Kim M., Proitsi P., Westwood S., Baird A., Nevado-Holgado A., Hye A., Bos I., Vos S.J.B., Vandenberghe R. (2019). A metabolite-based machine learning approach to diagnose Alzheimer-type dementia in blood: Results from the European Medical Information Framework for Alzheimer disease biomarker discovery cohort. Alzheimer’s Dement..

[56] Jack C.R., Knopman D.S., Jagust W.J., Shaw L.M., Aisen P.S., Weiner M.W., Petersen R.C., Trojanowski J.Q. (2010). Hypothetical model of dynamic biomarkers of the Alzheimer’s pathological cascade. Lancet Neurol..

[57] Mormino E.C., Betensky R.A., Hedden T., Schultz A.P., Amariglio R.E., Rentz D.M., Johnson K.A., Sperling R.A. (2014). Synergistic effect of beta-amyloid and neurodegeneration on cognitive decline in clinically normal individuals. JAMA Neurol..

[58] Tong T., Gao Q., Guerrero R., Ledig C., Chen L., Rueckert D., Initiative A.D.N. (2017). A Novel Grading Biomarker for the Prediction of Conversion From Mild Cognitive Impairment to Alzheimer’s Disease. IEEE Trans. Biomed. Eng..

[59] Cummings J. (2019). The National Institute on Aging-Alzheimer’s Association Framework on Alzheimer’s disease: Application to clinical trials. Alzheimer’s Dement..

[60] Allison S.L., Koscik R.L., Cary R.P., Jonaitis E.M., Rowley H.A., Chin N.A., Zetterberg H., Blennow K., Carlsson C.M., Asthana S. (2019). Comparison of different MRI-based morphometric estimates for defining neurodegeneration across the Alzheimer’s disease continuum. Neuroimage Clin..

[61] Young P.N.E., Estarellas M., Coomans E., Srikrishna M., Beaumont H., Maass A., Venkataraman A.V., Lissaman R., Jimenez D., Betts M.J. (2020). Imaging biomarkers in neurodegeneration: Current and future practices. Alzheimer’s Res..

[62] Farhan S., Fahiem M.A., Tauseef H. (2014). An ensemble-of-classifiers based approach for early diagnosis of Alzheimer’s disease: Classification using structural features of brain images. Comput. Math. Methods Med..

[63] Ahmed O.B., Mizotin M., Benois-Pineau J., Allard M., Catheline G., Ben Amar C., Alzheimer’s Disease Neuroimaging Initiative (2015). Alzheimer’s disease diagnosis on structural MR images using circular harmonic functions descriptors on hippocampus and posterior cingulate cortex. Comput. Med. Imaging Graph..

[64] Korolev S., Safiullin A., Belyaev M., Dodonova Y. Residual and plain convolutional neural networks for 3D brain MRI classification. Proceedings of the 2017 IEEE 14th International Symposium on Biomedical Imaging (ISBI 2017).

[65] Costafreda S.G., Dinov I.D., Tu Z., Shi Y., Liu C.Y., Kloszewska I., Mecocci P., Soininen H., Tsolaki M., Vellas B. (2011). Automated hippocampal shape analysis predicts the onset of dementia in mild cognitive impairment. Neuroimage.

[66] Lebedev A.V., Westman E., Van Westen G.J., Kramberger M.G., Lundervold A., Aarsland D., Soininen H., Kloszewska I., Mecocci P., Tsolaki M. (2014). Random Forest ensembles for detection and prediction of Alzheimer’s disease with a good between-cohort robustness. Neuroimage Clin..

[67] Spasov S., Passamonti L., Duggento A., Lio P., Toschi N., Alzheimer’s Disease Neuroimaging Initiative (2019). A parameter-efficient deep learning approach to predict conversion from mild cognitive impairment to Alzheimer’s disease. Neuroimage.

[68] Braak H., Braak E. (1991). Neuropathological stageing of Alzheimer-related changes. Acta Neuropathol..

[69] Gosche K.M., Mortimer J.A., Smith C.D., Markesbery W.R., Snowdon D.A. (2002). Hippocampal volume as an index of Alzheimer neuropathology: Findings from the Nun Study. Neurology.

[70] Gerardin E., Chetelat G., Chupin M., Cuingnet R., Desgranges B., Kim H.S., Niethammer M., Dubois B., Lehericy S., Garnero L. (2009). Multidimensional classification of hippocampal shape features discriminates Alzheimer’s disease and mild cognitive impairment from normal aging. Neuroimage.

[71] Chupin M., Gerardin E., Cuingnet R., Boutet C., Lemieux L., Lehericy S., Benali H., Garnero L., Colliot O., Alzheimer’s Disease Neuroimaging Initiative (2009). Fully automatic hippocampus segmentation and classification in Alzheimer’s disease and mild cognitive impairment applied on data from ADNI. Hippocampus.

[72] Vasta R., Augimeri A., Cerasa A., Nigro S., Gramigna V., Nonnis M., Rocca F., Zito G., Quattrone A., For The Alzheimer’s Disease Neuroimaging Initiative (2016). Hippocampal Subfield Atrophies in Converted and Not-Converted Mild Cognitive Impairments Patients by a Markov Random Fields Algorithm. Curr. Alzheimer Res..

[73] Rish I. An empirical study of the naive Bayes classifier. Proceedings of the IJCAI 2001 Workshop on Empirical Methods in Artificial Intelligence.

[74] Dickerson B.C., Bakkour A., Salat D.H., Feczko E., Pacheco J., Greve D.N., Grodstein F., Wright C.I., Blacker D., Rosas H.D. (2009). The cortical signature of Alzheimer’s disease: Regionally specific cortical thinning relates to symptom severity in very mild to mild AD dementia and is detectable in asymptomatic amyloid-positive individuals. Cereb. Cortex.

[75] Eskildsen S.F., Coupe P., Garcia-Lorenzo D., Fonov V., Pruessner J.C., Collins D.L., Alzheimer’s Disease Neuroimaging Initiative (2013). Prediction of Alzheimer’s disease in subjects with mild cognitive impairment from the ADNI cohort using patterns of cortical thinning. Neuroimage.

[76] Cho Y., Seong J.K., Jeong Y., Shin S.Y., Alzheimer’s Disease Neuroimaging Initiative (2012). Individual subject classification for Alzheimer’s disease based on incremental learning using a spatial frequency representation of cortical thickness data. Neuroimage.

[77] Huang L., Pan Z., Lu H. Automated diagnosis of Alzheimer’s disease with degenerate SVM-based adaboost. Proceedings of the 2013 5th International Conference on Intelligent Human-Machine Systems and Cybernetics (IHMSC).

[78] Raamana P.R., Weiner M.W., Wang L., Beg M.F., Alzheimer’s Disease Neuroimaging Initiative (2015). Thickness network features for prognostic applications in dementia. Neurobiol. Aging.

[79] Westman E., Simmons A., Zhang Y., Muehlboeck J.S., Tunnard C., Liu Y., Collins L., Evans A., Mecocci P., Vellas B. (2011). Multivariate analysis of MRI data for Alzheimer’s disease, mild cognitive impairment and healthy controls. Neuroimage.

[80] Coupe P., Eskildsen S.F., Manjon J.V., Fonov V.S., Collins D.L., Alzheimer’s Disease Neuroimaging Initiative (2012). Simultaneous segmentation and grading of anatomical structures for patient’s classification: Application to Alzheimer’s disease. Neuroimage.

[81] Lama R.K., Gwak J., Park J.S., Lee S.W. (2017). Diagnosis of Alzheimer’s Disease Based on Structural MRI Images Using a Regularized Extreme Learning Machine and PCA Features. J. Healthc. Eng..

[82] Zhao X., Ang C.K.E., Acharya U.R., Cheong K.H. (2021). Application of Artificial Intelligence techniques for the detection of Alzheimer’s disease using structural MRI images. Biocybern. Biomed. Eng..

[83] LeCun Y., Bengio Y., Hinton G. (2015). Deep learning. Nature.

[84] Payan A., Montana G. (2015). Predicting Alzheimer’s disease: A neuroimaging study with 3D convolutional neural networks. arXiv.

[85] Szegedy C., Liu W., Jia Y., Sermanet P., Reed S., Anguelov D., Erhan D., Vanhoucke V., Rabinovich A. Going deeper with convolutions. Proceedings of the IEEE Conference on Computer Vision and Pattern Recognition (CVPR).

[86] Russakovsky O., Deng J., Su H., Krause J., Satheesh S., Ma S., Huang Z., Karpathy A., Khosla A., Bernstein M. (2015). ImageNet Large Scale Visual Recognition Challenge. Int. J. Comput. Vis..

[87] Farooq A., Anwar S., Awais M., Rehman S. A deep CNN based multi-class classification of Alzheimer’s disease using MRI. Proceedings of the 2017 IEEE International Conference on Imaging Systems and Techniques (IST).

[88] Hosseini-Asl E., Ghazal M., Mahmoud A., Aslantas A., Shalaby A.M., Casanova M.F., Barnes G.N., Gimel’farb G., Keynton R., El-Baz A. (2018). Alzheimer’s disease diagnostics by a 3D deeply supervised adaptable convolutional network. Front. Biosci..

[89] Rokach L. (2010). Ensemble-based classifiers. Artif. Intell. Rev..

[90] Islam J., Zhang Y. (2018). Brain MRI analysis for Alzheimer’s disease diagnosis using an ensemble system of deep convolutional neural networks. Brain Inf..

[91] Wang H., Shen Y., Wang S., Xiao T., Deng L., Wang X., Zhao X. (2019). Ensemble of 3D densely connected convolutional network for diagnosis of mild cognitive impairment and Alzheimer’s disease. Neurocomputing.

[92] Shin H.C., Roth H.R., Gao M., Lu L., Xu Z., Nogues I., Yao J., Mollura D., Summers R.M. (2016). Deep Convolutional Neural Networks for Computer-Aided Detection: CNN Architectures, Dataset Characteristics and Transfer Learning. IEEE Trans. Med. Imaging.

[93] Hinton G.E., Osindero S., Teh Y.-W. (2006). A fast learning algorithm for deep belief nets. Neural Comput..

[94] Brosch T., Tam R., Alzheimer’s Disease Neuroimaging Initiative (2013). Manifold learning of brain MRIs by deep learning. Med. Image Comput. Comput. Assist. Interv..

[95] Yu W., Lei B., Ng M.K., Cheung A.C., Shen Y., Wang S. (2021). Tensorizing GAN With High-Order Pooling for Alzheimer’s Disease Assessment. IEEE Trans. Neural Netw. Learn. Syst..

[96] Lazli L., Boukadoum M., Mohamed O.A. (2020). A Survey on Computer-Aided Diagnosis of Brain Disorders through MRI Based on Machine Learning and Data Mining Methodologies with an Emphasis on Alzheimer Disease Diagnosis and the Contribution of the Multimodal Fusion. Appl. Sci..

[97] Doody R.S., Massman P., Dunn J.K. (2001). A method for estimating progression rates in Alzheimer disease. Arch. Neurol..

[98] Nagahama Y., Nabatame H., Okina T., Yamauchi H., Narita M., Fujimoto N., Murakami M., Fukuyama H., Matsuda M. (2003). Cerebral correlates of the progression rate of the cognitive decline in probable Alzheimer’s disease. Eur. Neurol..

[99] Barocco F., Spallazzi M., Concari L., Gardini S., Pelosi A., Caffarra P. (2017). The Progression of Alzheimer’s Disease: Are Fast Decliners Really Fast? A Four-Year Follow-Up. J. Alzheimer’s Dis..

[100] Edwin T.H., Strand B.H., Persson K., Engedal K., Selbaek G., Knapskog A.B. (2021). Trajectories and risk factors of dementia progression: A memory clinic cohort followed up to 3 years from diagnosis. Int. Psychogeriatr..

[101] Deaton A., Cartwright N. (2018). Understanding and misunderstanding randomized controlled trials. Soc. Sci. Med..

[102] Jutten R.J., Sikkes S.A.M., Van der Flier W.M., Scheltens P., Visser P.J., Tijms B.M., Alzheimer’s Disease Neuroimaging Initiative (2021). Finding Treatment Effects in Alzheimer Trials in the Face of Disease Progression Heterogeneity. Neurology.

[103] Cummings J.L., Morstorf T., Zhong K. (2014). Alzheimer’s disease drug-development pipeline: Few candidates, frequent failures. Alzheimer’s Res..

[104] Tolar M., Abushakra S., Hey J.A., Porsteinsson A., Sabbagh M. (2020). Aducanumab, gantenerumab, BAN2401, and ALZ-801-the first wave of amyloid-targeting drugs for Alzheimer’s disease with potential for near term approval. Alzheimer’s Res..

[105] Jang H., Park J., Woo S., Kim S., Kim H.J., Na D.L., Lockhart S.N., Kim Y., Kim K.W., Cho S.H. (2019). Prediction of fast decline in amyloid positive mild cognitive impairment patients using multimodal biomarkers. Neuroimage Clin..

[106] Sadiq M.U., Kwak K., Dayan E., Alzheimer’s Disease Neuroimaging Initiative (2022). Model-based stratification of progression along the Alzheimer disease continuum highlights the centrality of biomarker synergies. Alzheimer’s Res..

[107] Petitjean F., Ketterlin A., Gançarski P. (2011). A global averaging method for dynamic time warping, with applications to clustering. Pattern Recognit..

[108] Ward Jr J.H. (1963). Hierarchical grouping to optimize an objective function. J. Am. Stat. Assoc..

[109] Gamberger D., Lavrac N., Srivatsa S., Tanzi R.E., Doraiswamy P.M. (2017). Identification of clusters of rapid and slow decliners among subjects at risk for Alzheimer’s disease. Sci. Rep..

[110] Karkkainen M., Prakash M., Zare M., Tohka J., For The Alzheimer’s Disease Neuroimaging Initiative (2020). Structural Brain Imaging Phenotypes of Mild Cognitive Impairment (MCI) and Alzheimer’s Disease (AD) Found by Hierarchical Clustering. Int. J. Alzheimer’s Dis..

[111] Lee J.S., Kim C., Shin J.H., Cho H., Shin D.S., Kim N., Kim H.J., Kim Y., Lockhart S.N., Na D.L. (2018). Machine Learning-based Individual Assessment of Cortical Atrophy Pattern in Alzheimer’s Disease Spectrum: Development of the Classifier and Longitudinal Evaluation. Sci. Rep..

[112] Fisher C.K., Smith A.M., Walsh J.R., Coalition Against Major Diseases, and Abbott, Alliance for Aging Research, Alzheimer’s Association, Alzheimer’s Foundation of America, AstraZeneca Pharmaceuticals LP, Bristol-Myers Squibb Company, Critical Path Institute, CHDI Foundation, Inc. (2019). Machine learning for comprehensive forecasting of Alzheimer’s Disease progression. Sci. Rep..

[113] Mnih V., Larochelle H., Hinton G.E. (2012). Conditional Restricted Boltzmann Machines for Structured Output Prediction. arXiv.

[114] Thalhauser C.J., Komarova N.L. (2012). Alzheimer’s disease: Rapid and slow progression. J. R. Soc. Interface.

[115] Reith F.H., Mormino E.C., Zaharchuk G. (2021). Predicting future amyloid biomarkers in dementia patients with machine learning to improve clinical trial patient selection. Alzheimer’s Dement..

[116] He K., Zhang X., Ren S., Sun J. Deep residual learning for image recognition. Proceedings of the 2016 IEEE Conference on Computer Vision and Pattern Recognition (CVPR).

[117] Zhou Y., Wang F., Tang J., Nussinov R., Cheng F. (2020). Artificial intelligence in COVID-19 drug repurposing. Lancet Digit. Health.

[118] Ying R., Bourgeois D., You J., Zitnik M., Leskovec J. (2019). Gnnexplainer: Generating explanations for graph neural networks. Adv. Neural Inf. Process. Syst..

[119] Goerdten J., Cukic I., Danso S.O., Carriere I., Muniz-Terrera G. (2019). Statistical methods for dementia risk prediction and recommendations for future work: A systematic review. Alzheimer’s Dement..

[120] Rouquette A., Falissard B. (2011). Sample size requirements for the internal validation of psychiatric scales. Int. J. Methods Psychiatr. Res..

[121] Ma J., Yu M.K., Fong S., Ono K., Sage E., Demchak B., Sharan R., Ideker T. (2018). Using deep learning to model the hierarchical structure and function of a cell. Nat. Methods.

[122] Sancesario G.M., Bernardini S. (2018). Alzheimer’s disease in the omics era. Clin. Biochem..

